# Model-Based Estimation of Ankle Joint Stiffness

**DOI:** 10.3390/s17040713

**Published:** 2017-03-29

**Authors:** Berno J. E. Misgeld, Tony Zhang, Markus J. Lüken, Steffen Leonhardt

**Affiliations:** Philips Chair for Medical Information Technology, RWTH Aachen University, Pauwelsstrasse 20, 52074 Aachen, Germany; tony.zhang@rwth-aachen.de (T.Z.); lueken@hia.rwth-aachen.de (M.J.L.); leonhardt@hia.rwth-aachen.de (S.L.)

**Keywords:** joint stiffness estimation, body-worn sensors, magnetic, angular rate and gravity sensors, BSN

## Abstract

We address the estimation of biomechanical parameters with wearable measurement technologies. In particular, we focus on the estimation of sagittal plane ankle joint stiffness in dorsiflexion/plantar flexion. For this estimation, a novel nonlinear biomechanical model of the lower leg was formulated that is driven by electromyographic signals. The model incorporates a two-dimensional kinematic description in the sagittal plane for the calculation of muscle lever arms and torques. To reduce estimation errors due to model uncertainties, a filtering algorithm is necessary that employs segmental orientation sensor measurements. Because of the model’s inherent nonlinearities and nonsmooth dynamics, a square-root cubature Kalman filter was developed. The performance of the novel estimation approach was evaluated in silico and in an experimental procedure. The experimental study was conducted with body-worn sensors and a test-bench that was specifically designed to obtain reference angle and torque measurements for a single joint. Results show that the filter is able to reconstruct joint angle positions, velocities and torque, as well as, joint stiffness during experimental test bench movements.

## 1. Introduction

An accurate real-time estimation of human joint stiffness would be beneficial in many situations. For example, active prosthetics, orthotics, or exoskeletons could use information on quantitative joint stiffness to adjust their control strategies [1]. Currently, such systems are increasingly fitted with physically adjustable elements [2]. Therefore, the adjustment of, for example, a physically controllable interaction stiffness in robotic support is a possible area of application. Besides these movement support applications, time-resolved knowledge of human joint stiffness can be useful in motion analysis or as a detection system for pathologic movement states, such as spasticity [3].

The stiffness of human lower extremity joints changes naturally during locomotion. This phenomenon is typically called *stiffness modulation* and serves as an involuntary adaptation of the neuromusculoskeletal system to handle contact situations in a highly uncertain environment. Stiffness modulation is suggested to increase gait stability, or absorb shocks [4]. Although it can be argued that stiffness is the major component that is changed during human locomotion, other passive elements (such as viscous damping) also exist. Together with inertia, joint dynamic properties can be generalised to the concept of mechanical impedance. A mechanical impedance can be regarded as a frequency dependent and (for most human joints) nonlinear mechanical resistance to excitation of a force (torque) or a velocity (angular rate). Since its recognition in locomotion in the 1980s [5], mechanical impedance has been well researched and is suggested to play a major role in the stabilisation of unstable walking dynamics and motion learning [4,6,7]. However, this particular study focuses on the estimation of joint stiffness, which is a major component of joint impedance.

Different dynamic modelling and estimation approaches for the ankle joint have been reported. Early publications focused mainly on exploration of the so-called *quasi-stiffness*, which is defined as the derivative of the torque with respect to the angle [8]. For instance, Kearny and Hunter [9] presented the estimation of dynamic stiffness transfer functions from experimental measurements of the ankle; they concluded that a linear modelling approach is not enough to account for ankle stiffness nonlinearities. In contrast, early models of the lower extremities aim (for example) for the estimation of joint torques, or calculation of stress rates, or analysis of human movement [10,11,12]. This was followed by more complex models of the lower extremities, that were extended to the three-dimensional case and contained active muscle models that were driven by electromyographic (EMG) measurements [13,14]. However, the linear and nonlinear modelling of single isolated joints continued to attract interest of researchers. For example, Riener and Edrich examined passive elastic joint moments for knee and ankle joint [15]. Also, in a test scenario, Lee et al. presented a linear multivariable analysis on dynamic ankle stiffness for active muscles for a two degrees-of-freedom (DOF) ankle joint [16]. Various estimation procedures (e.g., linear parameter varying and nonlinear estimation) are available in the literature. Procedures for the modelling of isolated human joints are presented, for example, in [17,18]. In contrast, however, very few forward-driven dynamic models that address isolated joints are available in the literature. Nevertheless, one such example was presented by Sartori et al., in which the model is driven by EMG data and calibrated to satisfy joint moments about a single DOF joint [19]. Another example is an EMG-driven musculoskeletal model that is used to estimate muscle forces and knee joint movement [20]. The advantage of EMG-driven models is that they can predict joint stiffness changes that occur due to a muscle coactivation. Changes in muscle activation and muscle-tendon-complex (MTC) kinematics result in changes in MTC stiffness, which is reflected at the level of the joint [21]. Since the tendons show nonlinear characteristics of force over displacement, a coactivation of antagonistic muscles leads to a change of overall joint stiffness. Another advantage of EMG-driven neuromusculoskeletal models is that they are able to take into account different muscle activation patterns, even when the external kinematics and joint moments are the same. These were reported in static tasks which require force and position control [22]. Examples of models to be used in joint stiffness estimation were published by Pfeifer et al. [23] for the human knee and by Rouse et al. [24] for the human ankle. However, both models are forward simulations and the model presented in [24] does not incorporate EMG information.

In this study, we present a model-based approach aiming at stiffness estimation to be used in, for example, ankle orthotic applications [25,26]. To minimise model errors that occur in forward estimation procedures, our approach employs a nonlinear observer. To our knowledge, no such approach for the isolated ankle joint has yet been published. Our observer employs a nonlinear biomechanical model, surface EMG (sEMG) measurements, and joint angle measurements. Since nonsmooth model dynamics prevent classical observer designs that employ Jacobian linearisation techniques, we employ a square-root cubature Kalman filter (SCKF) [27]. Moreover, the SCKF was chosen as it can handle strong model-inherent nonlinearities and model uncertainties. Since the model takes antagonistic muscles into account, the stiffness estimation is able to reconstruct ankle stiffness changes due to muscle coactivation effects.

This paper is organised as follows: Section 2 formulates a new model for the lower extremity in the sagittal plane and Section 3 presents the model-based filtering approach and the experimental setup. Simulation and experimental results of the procedure are described in Section 4, and Section 5 presents the conclusion and discussion.

## 2. Dynamic Lower Extremity Modelling

The model of the lower extremity is based on the sagittal plane kinematics of the knee joint that was presented in [28]. However, the main focus of the present study is the extension of this model with ankle and hip dynamic models. The human ankle joint consists of an upper (*articulatio talocruralis*; TC) and a lower (*articulatio subtalaris* and *articulatio talocalcaneonavicularis*) part. This study focusses on the modelling of dorsiflexion and plantar flexion, which is mainly achieved by the TC [29,30,31]. Although the TC is generally considered as a one-degree-of-freedom joint disagreement exists about the rotation axis/axes. Early studies indicated two discrete axes of rotation [32,33], one at dorsiflexion and one at plantar flexion. However, a subsequent study identified a single, fixed axis of rotation [34]. More recent research considered a continuously shifting axis of rotation with foot movement [29,30,35]. Nonetheless, some studies support the approach of a single fixed axis [31,36]. Moreover, the modelling approach with a single hinge joint was successfully employed in several studies that yielded plausible results [37,38,39,40,41]. Consequently, in the present study we model the TC as a simple hinge joint.

### 2.1. Ankle Kinematic Model

**Figure 1:**
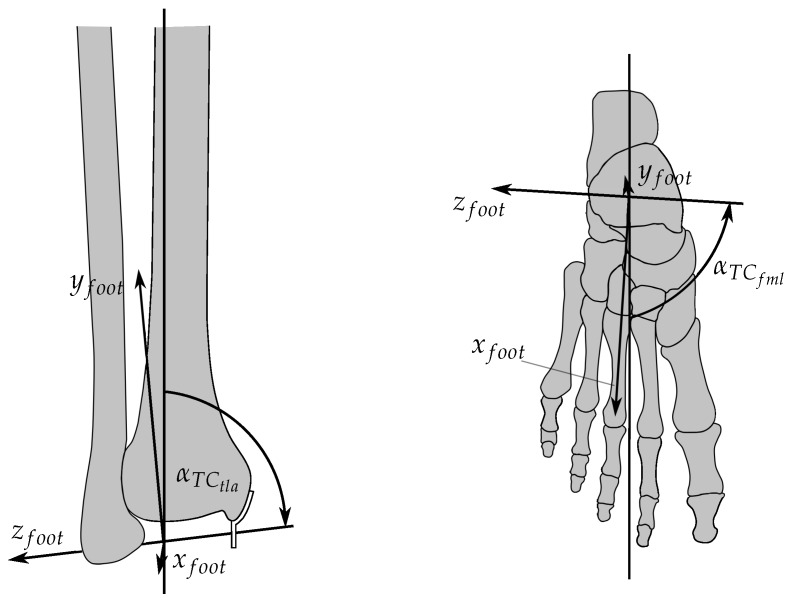
Rotation axis of the TC and local body-fixed coordinate system of the foot. **Left**: View at the coordinate system from the front; $\alpha_{TC_{tla}}$ is the angle between tibia axis and rotation axis. **Right**: View at the coordinate system from above; $\alpha_{TC_{fml}}$ is the angle between mid-line of the foot and the rotation axis.

The local foot coordinate system is set-up with respect to the knee reference system, where the latter is (later on) related to the global reference system located at the hip. The knee reference coordinate system is located at the contact points between condyles and tibia plateau at a knee angle of $0^{\circ }$. Thus, the y-axis $y_{knee}$ points along the tibia, whereas $x_{knee}$ is anterograde at upright standing. $z_{knee}$ is chosen so as to span a right-handed coordinate system. A local coordinate system is defined for the rotation of the foot, which is body-fixed to the foot. However, this allows to rotate the foot with respect to the global coordinate system. Figure 1 shows the local foot coordinate system, which rotates about the z-axis $z_{ankle}$. The inclination and orientation of $z_{ankle}$ were determined according to the results of [34].

**Figure 2:**
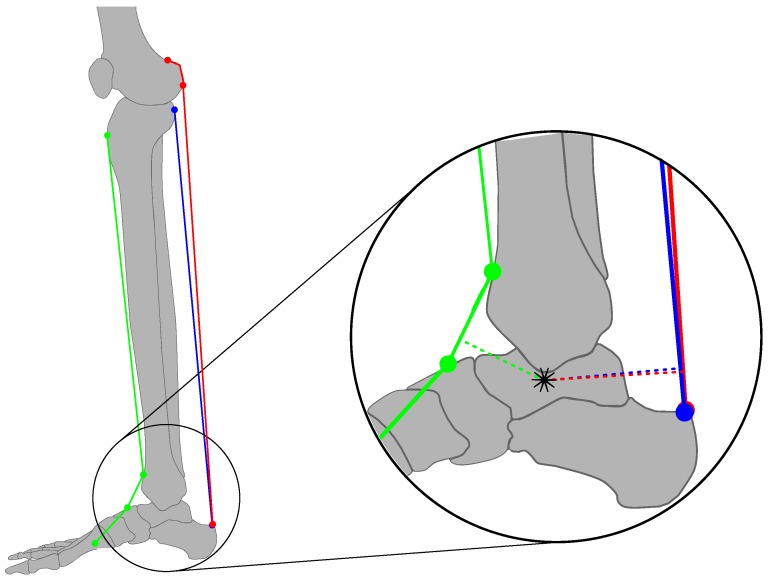
Ankle joint model (green: TA, blue: SOL, red: GAS). Dotted lines illustrate the lever arms; the star indicates the variable centre of rotation that is obtained by projection into the sagittal plane. TA, musculus tibialis anterior; SOL, musculus soleus; GAS, musculus gastrocnemius.

The origin of the foot coordinate system was placed between both *malleoli*. The local x-axis $x_{foot}$ is perpendicular to $z_{foot}$ and parallel to the foot sole. $y_{foot}$ is aligned so as to span a right-handed coordinate system. The ankle joint angle $\varphi_{ankle}$ is defined by a rotation of the foot coordinate system around $z_{foot}$ with respect to the global *y*-axis. Furthermore, the zero position $\varphi_{ankle}=0^{\circ }$ is defined at upright standing ($y_{knee}$ is perpendicular to $x_{ankle}$). The angular range of the ankle joint was defined to lie on the interval $-60^{\circ }<\varphi_{ankle}<20^{\circ }$. The resulting transformation of a point $p^{foot}$ in the local coordinate system of the foot is defined in homogeneous coordinates with help of the homogeneous transformation matrix $T(\varphi_{ankle})$ To set-up rotation matrices, $x_{trans}$ and $y_{trans}$ describe the origin of the foot coordinate system in the knee coordinate system. Moreover, the rotation angles are defined as $\beta =\alpha_{TC_{fml}}-90^{\circ }$ and $\gamma =90^{\circ }-\alpha_{TC_{tla}}$. For actuation of the ankle joint, kinematic considerations are made for three main muscles: the *musculus tibialis anterior* (TA) as a primary actuator for dorsiflexion, as well as the *musculus soleus* (SOL) and the *musculus gastrocnemius* (GAS) as the primary actuators for plantar flexion [42]. The reasons for this decision were: a) to have reliable sEMG signals in the filter, and b) to limit the dimension of the resulting state-space model (each muscle increases the filter order by 2.) Figure 2 is an overview of the muscle kinematics used to calculate the lever arms.

The TA originates from the lateral condyle of the tibia and sets on along the inside of the foot. Figure 2 shows that the TA is guided by two medially aligned ligaments (*retinaculum musculorum extensorum superius* and *inferius*). The GAS (Note that the GAS model was scaled accordingly, to account for the medial and lateral part simultaneously) originates from its two heads at the condyles of the femur where, in case of the SOL, the origin is the posterior-proximal end of the tibia and fibula. Both muscles are joined in the Achilles tendon which inserts into the posterior surface of the heel bone (*calcaneus*). As described in [28], the muscles are approximated by line segments from origin to fixation point. The fixation of the TA by means of the *retinacula* is realised by two nodal points for determination of the effective origin and point of action. These two points were taken from the literature for the shank and for the foot [43,44], respectively (Figure 2). From the position of the TC rotation axis and the coordinates of the nodal point, the length of the MTC $l_{MT,i}$ and the lever arms $r_{ma,i}$ can be calculated. The length $l_{MT,i}$ is a result of the sum of the single line segment. The lever arms are defined as the distance of the rotation axis to the effective direction of the muscle force. The effective direction lies on the straight line

(1) $$g:\;x={\hat{p}}_{O,i}^{global}+\lambda \left({\hat{p}}_{I,i}^{global}-{\hat{p}}_{O,i}^{global}\right),$$

where λ is the variable of the straight line equation and ${\hat{p}}_{O,i}^{global}$ and ${\hat{p}}_{I,i}^{global}$ denote global coordinates of the projection of the origin and the contact point of the muscle into the sagittal plane. Hence, the lever arm of the muscle *i* results in

(2) $$r_{ma,i}=\frac{|\left({\hat{p}}_{IC}^{global}-{\hat{p}}_{O,i}^{global}\right)\times \left({\hat{p}}_{I,i}^{global}-{\hat{p}}_{O,i}^{global}\right)|}{|{\hat{p}}_{I,i}^{global}-{\hat{p}}_{O,i}^{global}|}.$$

**Figure 3:**
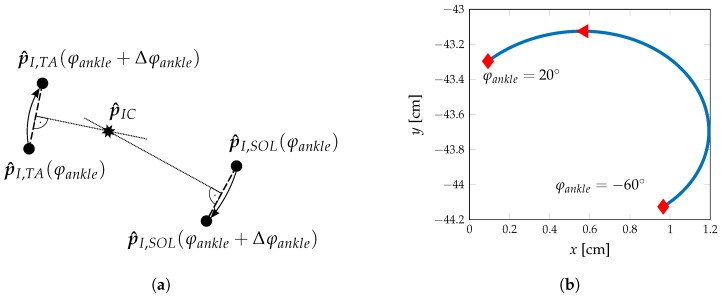
Determination of the current centre-of-rotation in the sagittal plane. (**a**) Determination of the current centre-of-rotation by a small angle change $\Delta \varphi_{ankle}$. ${\hat{p}}_{IC}$ results from the intersection of the perpendicular lines bisecting straight lines connecting $p_{I,i}(\varphi_{ankle})$ and $p_{I,i}(\varphi_{ankle}+\Delta \varphi_{ankle})$. (**b**) Change of current centre-of-rotation with $\varphi_{ankle}$ with projection into the sagittal plane.

In Equation (2), ${\hat{p}}_{IC}^{global}$ represents the global coordinates of the current rotation axis. Due to the projection into the sagittal plane, a resulting movement of rotation axis can be observed and is visualised in Figure 3a,b.

Note that if the muscle has an effective origin or contact point, these are used to determine the effective direction. This is, for example, the case for the TA, where the effective origin is in the shank and the effective contact point is in the foot. In addition, the GAS has an effective origin at the condyles of the femur given the corresponding knee alignment. Moreover, the GAS is a biarticulate muscle acting on knee and ankle joint at the same time and, as such, the corresponding variables depend on the orientation of the ankle and knee joint angles.

Line segment lengths and lever arms of the modelled kinematics were calculated offline over the whole range of ankle angles $\varphi_{ankle}$ and knee angles $\varphi_{knee}$ at increments of $\Delta \varphi_{angle}=1^{\circ }$ and approximated by fourth-order polynomials. The muscle contraction speed $v_{M}$ can then be calculated from angular velocity and time-derivative of $l_{MT}\left(\varphi_{ankle}\right)$. For TA and SOL, the time-derivative of $l_{MT}\left(\varphi_{ankle}\right)$ results in

(3) $$\begin{array}{cc} v_{M} & =\frac{dl_{M}}{dt}=\frac{d}{dt}\left(\frac{l_{MT}\left(\varphi_{ankle}\right)-l_{T}}{\cos\alpha_{p}}\right)\\ & =\frac{d\varphi_{ankle}}{dt}\frac{d}{d\varphi_{ankle}}\left(\frac{l_{MT}\left(\varphi_{ankle}\right)-l_{T}}{\cos\alpha_{p}}\right)\\ & ={\dot{\varphi }}_{ankle}\frac{dl_{MT}}{d\varphi_{ankle}}\frac{1}{\cos\alpha_{p}}, \end{array}$$

with muscle length $l_{M}$ and pennation angle $\alpha_{p}$. As for the GAS we obtain

(4) $$\begin{array}{cc} v_{M} & =\frac{dl_{M}}{dt}=\frac{d}{dt}\left(\frac{l_{MT}\left(\varphi_{ankle},\varphi_{knee}\right)-l_{T}}{\cos\alpha_{p}}\right)\\ & =\frac{d\varphi_{ankle}}{dt}\frac{\partial }{\partial \varphi_{ankle}}\left(\frac{l_{MT}\left(\varphi_{ankle},\varphi_{knee}\right)-l_{T}}{\cos\alpha_{p}}\right)+\frac{d\varphi_{knee}}{dt}\frac{\partial }{\partial \varphi_{knee}}\left(\frac{l_{MT}\left(\varphi_{ankle},\varphi_{knee}\right)-l_{T}}{\cos\alpha_{p}}\right)\\ & =\left({\dot{\varphi }}_{ankle}\frac{\partial l_{MT}}{\partial \varphi_{ankle}}+{\dot{\varphi }}_{knee}\frac{\partial l_{MT}}{\partial \varphi_{knee}}\right)\frac{1}{\cos\alpha_{p}}. \end{array}$$

Note that in Equations (3) and (4) it is assumed that the tendon length $l_{T}$ is constant. However, whereas taking the Achilles tendon strain into account is important to determine joint stiffness, computational simplifications are necessary to make the model treatable.

### 2.2. Hip Kinematic Model

The hip joint is typically modelled as a 3 DOF ball joint [45] or a 1 DOF hinge joint in the sagittal plane [43]. For the purpose of the present model, only the influence of the sagittal plane orientation of the hip is considered. Hence, the hip joint is modelled as a hinge joint. The MTC length of the biarticular muscles that act on the knee and hip joint is influenced by the orientation of the hip joint. For further simplification, the influence of hip abduction/adduction and outer/inner rotation is considered to be small as compared to hip flexion/extension and is subsequently neglected. Biarticular muscle groups that are considered in the knee model presented by Misgeld et al. [28] are the *musculus quadriceps femoris* (QF) and the *ischiocrural muscles* (HAMS). The lengths of their corresponding MTCs $l_{MT,QF}$ and $l_{MT,HAMS}$ are influenced by the hip angle $\varphi_{hip}$. The hip angle $\varphi_{hip}$ is defined as the angle between the pelvis and proximal extension of the thigh; it is zero in upright standing and has a range of $-20^{\circ }<\varphi_{hip}<150^{\circ }$, where $\varphi_{hip}>0^{\circ }$ is defined to be flexion. The hip angle is considered in an analogous way for the GAS calculations.

### 2.3. Lower Extremity Dynamics

**Figure 4:**
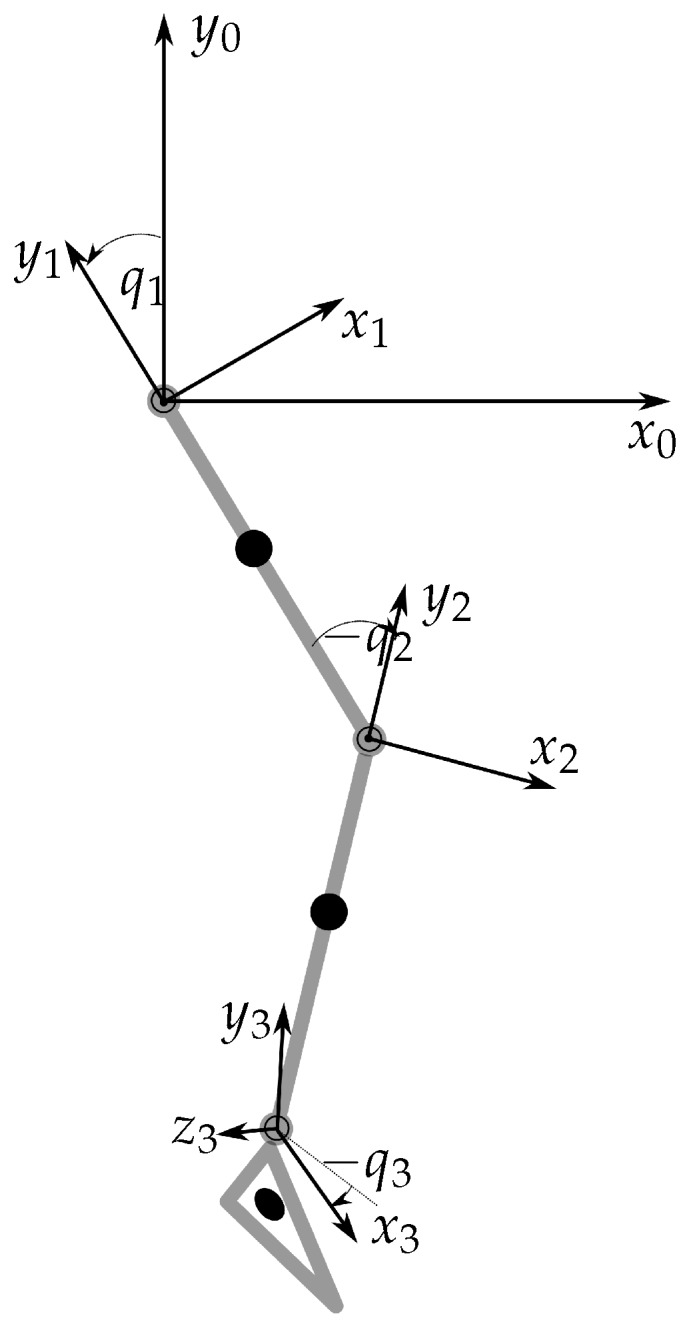
Dynamic lower extremity model. System 0: global coordinate system; system 1: reference frame thigh; system 2: reference frame shank; system 3: reference frame foot ($z_{3}$ is the rotation axis of the TC).

The dynamic model of the lower extremity follows the approach of Yamaguchi and Zajac [46]. In contrast to [46], in our model the ankle joint TC rotation axis is not perpendicular to the sagittal plane; moreover, other coordinate systems and angles are used. The individual determination of the centre-of-mass and centre-of-inertia is based on the relative data of de Leva [47]. The kinematics of the knee joint are based on [28], which assumes a fixed centre-of-rotation for the dynamics; this is in contrast to the knee kinematics, where the translation of the centre-of-rotation is still considered. The present model consists of three segments and three hinge joints (Figure 4).

The derivation of the multibody dynamics follows the Euler-Lagrange equation approach. On the one hand, elastic and viscous joint properties are modelled as external forces. On the other, the potential energy consists of segmental mass $m_{i}$ in the corresponding centre-of-gravity, and the total kinetic energy results from the kinetic energy of the three modelled segments. Substitution of potential and kinetic energy into the Lagrangian functional and subsequent partial differentiation leads to the Euler-Lagrange equations

(5) $$M\left(q\right)\ddot{q}+D\left(q,\dot{q}\right)\dot{q}+k\left(q\right)=\tau ,$$

where M(q) denotes the symmetric positive definite inertia matrix, q is the vector of generalised coordinates, and τ denotes the vector of external forces and torques acting on the lower extremity model. The matrix $D(q,\dot{q})$ contains centrifugal and Coriolis terms and is defined as

(6) $$d_{kj}=\sum_{i=1}^{3}\frac{1}{2}\left\{\frac{\partial m_{kj}}{\partial q_{j}}+\frac{\partial m_{ki}}{\partial q_{j}}-\frac{\partial m_{ij}}{\partial q_{k}}\right\}\dot{q_{i}}.$$

The last term in Equation (5) relates to potential energy and is defined as

(7) $$k_{i}=\frac{\partial P}{\partial q_{i}}.$$

In order to determine the complete dynamics, the external joint torques are required, these external torques act on the dynamic system (5) through τ. However, for the hip joint no external torques are taken into account, because the hip joint is only considered for its influence on the length of the corresponding MTC that act on the knee joint. The corresponding external torque for the hip joint, $\tau_{1}$, is unknown. Consequently, the corresponding hip angle $q_{1}$ is considered as an external input signal. The equations of motion, given by Equation (5), have to be reduced with respect to the two unknowns $q_{2}$ and $q_{3}$:

(8) $$\hat{M}\left(\hat{q},q_{1}\right)\ddot{\hat{q}}+\hat{D}\left(\hat{q},\dot{\hat{q}},q_{1},\dot{q_{1}}\right)\dot{\hat{q}}+\hat{k}\left(\hat{q},q_{1}\right)=\hat{\tau }\left(\hat{q},\dot{\hat{q}},q_{1},\dot{q_{1}},\ddot{q_{1}}\right),$$

with

(9) $$\begin{array}{cc} \hat{q} & =\left[\begin{array}{c} q_{2}\\ q_{3} \end{array}\right],\;\hat{M}=\left[\begin{array}{cc} m_{22} & m_{23}\\ m_{32} & m_{33} \end{array}\right],\;\hat{D}=\left[\begin{array}{cc} d_{22} & d_{23}\\ d_{32} & d_{33} \end{array}\right],\;\hat{k}=\left[\begin{array}{c} k_{2}\\ k_{3} \end{array}\right],\\ \hat{\tau } & =\left[\begin{array}{c} \tau_{2}\\ \tau_{3} \end{array}\right]-\ddot{q_{1}}\left[\begin{array}{c} m_{21}\\ m_{31} \end{array}\right]-\dot{q_{1}}\left[\begin{array}{c} d_{21}\\ d_{31} \end{array}\right], \end{array}$$

which is an equivalent representation with respect to the two lower rows of Equation (5). The external joint torques $\tau_{j}$ consist of actively generated joint torques $\tau_{j}^{T}$, passively generated joint torques $\tau_{j}^{E}$ and $\tau_{j}^{V}$ as well as externally generated torques $\tau_{j}^{ext}$ like, for example, ground reaction forces. These torques are combined as

(10) $$\tau_{j}=\tau_{j}^{T}+\tau_{j}^{E}+\tau_{j}^{V}+\tau_{j}^{ext}.$$

The active torques for each joint are considered as the sum of all active contributions $\tau_{i,j}^{T}$ of the involved muscles *i* acting on the considered joint *j*

(11) $$\tau_{j}^{T}=\sum_{i=1}^{N_{j}}\tau_{i,j}^{T}=\sum_{i=1}^{N_{j}}r_{ma_{i,j}}F_{i}^{T},$$

where $N_{j}$ is the number of muscles that act on the joint *j*, $r_{ma_{i,j}}$ is the lever arm of the corresponding muscle that acts on joint *j* and $F_{i}^{T}$ is the tendon force that is calculated from the MTC dynamic models. These include an activation dynamics model and a modified extended Hill-model; these two dynamic models are not repeated here as they are extensively described in [28]. The inclusion of the passively generated joint torques is based on a simplification of the extended Hill model [28,48]. For that, the tendons are assumed to be stiff elements. Therefore, for each single muscle the parallel elastic and viscous element can be separated from the contractile element and combined into a single elastic and a single viscous element for each joint, that represents the combined viscoelastic torques around that joint. The viscous torques $\tau_{j}^{V}$ are described by [49]

(12) $$\tau_{j}^{V}=K_{V_{j}}\mathrm{sgn}\left(\dot{q_{j}}\right){\left|\dot{q_{j}}\right|}^{n_{V_{j}}},$$

with damping constant $K_{V_{j}}$ for the joint *j*. The modelling of elastic moment of the knee joint is based on [15] and is given as

(13) $$\tau_{2}^{E}=e^{a_{1_{k}}+b_{1_{k}}q_{1}+c_{1_{k}}q_{2}+d_{1_{k}}q_{3}}-e^{a_{2_{k}}+b_{2_{k}}q_{1}+c_{2_{k}}q_{2}+d_{2_{k}}q_{3}}+e_{k}-\tau_{k}^{*},$$

where the additional term $\tau_{k}^{*}$ accounts for the steepest increase of the elastic torque at full knee extension:

(14) $$\tau_{k}^{*}=e^{f_{k}+g_{k}q_{2}}.$$

The elastic torque at the ankle joint is modelled by the following form

(15) $$\tau_{3}^{E}=e^{a_{1_{a}}+b_{1_{a}}q_{2}+c_{1_{a}}q_{3}}-e^{a_{2_{a}}+b_{2_{a}}q_{2}+c_{2_{a}}q_{3}}+d_{a}.$$

**Figure 5:**
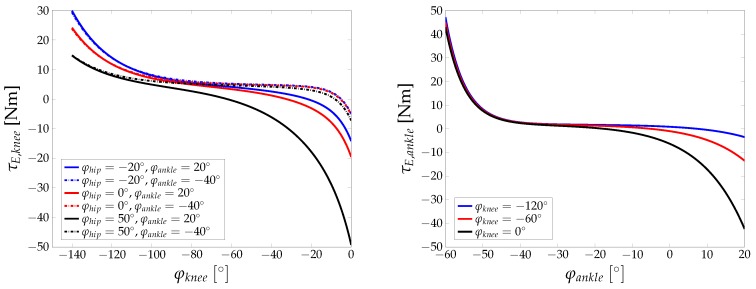
Passive elastic joint torques for knee (**left**) and ankle (**right**) joint, based on parameters presented in [15].

The nonlinear elastic torques for knee and ankle joint are shown in Figure 5. The dependency of the joint torques on the alignment of neighbouring joints is clearly shown and is due to the biarticular nature of the muscle groups involved.

Finally, ground reaction forces (GRF) have to be considered in case the lower extremity has, for example, contact with the ground. Assuming the point of action $p_{GRF}$ is known, the components of the GRF vector can easily be translated to model torques

(16) $$\tau_{j}^{ext}=\left(\left(p_{GRF}-o_{j}\right)\times f_{GRF}\right)e_{z_{j}},$$

where $o_{j}$ denotes the joint centre and $e_{z_{j}}$ is defined as the unit vector in the direction of the rotation axis of the joint *j*. The model is extended with muscle activation dynamics [28,50] and muscle contraction dynamics of an extended Hill type [28,51]. For the total of six muscles (RF, ST, GAS, VM, SOL, TA) that are included in the model, the state vector $\tilde{x}$ is used to describe activation and contraction dynamics. Denoting $x_{1}=q_{2}=\varphi_{knee}$, $x_{2}=q_{3}=\varphi_{ankle}$, $x_{3}={\dot{\varphi }}_{knee}$ and $x_{4}={\dot{\varphi }}_{ankle}$, the dynamic model of the lower extremity can be given in the form of a state-space model as

(17) $$\begin{array}{cc} {\dot{x}}_{1} & =x_{3}\\ {\dot{x}}_{2} & =x_{4}\\ {}[{\dot{x}}_{3},{\dot{x}}_{4}{]}^{T} & ={\hat{M}}^{-1}\left(x,u\right)\left\{\hat{\tau }\left(x,u\right)-\hat{k}\left(x,u\right)-\hat{D}\left(x,u\right){\left[x_{3},x_{4}\right]}^{T}\right\}\\ \dot{\tilde{x}} & =\tilde{f}\left(\tilde{x},u\right). \end{array}$$

**Figure 6:**
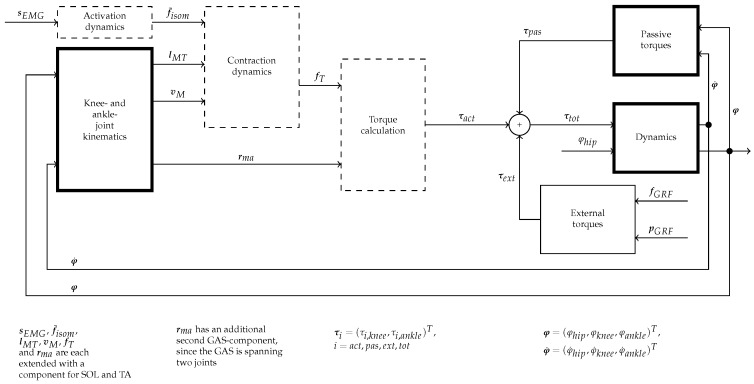
Block diagram of the lower extremity dynamic model. The *dashed* boxes are unchanged compared to the model presented in [28], expcept for the additional muscles (SOL, TA). The *bold* boxes are fundamentally extended or new.

In Equation (17), $x={\left[x_{1},x_{2},x_{3},x_{4},{\tilde{x}}^{T}\right]}^{T}\in R^{16}$ denotes the state vector and $u\in R^{13}$ is the input vector, consisting of sEMG-signals, hip angle $\varphi_{hip}$, components of GRF $f_{GRF}$ and three coordinates of point of action of the force $p_{GRF}$. Thus, the full state vector is given by

(18) $$\begin{array}{c} x=[\varphi_{knee},\varphi_{ankle},{\dot{\varphi }}_{knee},{\dot{\varphi }}_{ankle},a_{RF},a_{HAMS},a_{VM},a_{GAS},a_{SOL},a_{TA},\\ {\bar{F}}_{isom_{RF}},{\bar{F}}_{isom_{HAMS}},{\bar{F}}_{isom_{VM}},{\bar{F}}_{isom_{GAS}},{\bar{F}}_{isom_{SOL}},{\bar{F}}_{isom_{TA}}{]}^{T} \end{array}$$

where $a_{i}$ is the activation of muscle *i* and ${\bar{F}}_{isom_{i}}$ is the isometric force of muscle *i*, normalised with the maximal isometric force of muscle *i*. The input vector is given by

(19) $$u={\left[s_{RF},s_{HAMS},s_{VM},s_{GAS},s_{SOL},s_{TA},\varphi_{hip},{\dot{\varphi }}_{hip},{\ddot{\varphi }}_{hip},x_{CoP},y_{CoP},z_{CoP},F_{{GRF}_{x}},F_{{GRF}_{y}},F_{{GRF}_{z}}\right]}^{T}$$

where $s_{i}$ is the rectified, normalised and filtered sEMG raw signal from muscle *i*, $x_{CoP},y_{CoP},z_{CoP}$ are the coordinates of the centre of pressure, and $F_{{GRF}_{x}},F_{{GRF}_{y}},F_{{GRF}_{z}}$ are the components of the ground reaction force. Moreover, $\tilde{f}\left(\cdot \right):R^{12}\times R^{12}\mapsto R^{12}$ denotes the nonlinear activation and contraction dynamics [28]. The output equation consists of joint angle measurements $y={\left[x_{1},x_{2}\right]}^{T}\in R^{2}$. The state-space model given by Equation (17) can be reformulated in a compact form as

(20) $$\begin{array}{cc} \dot{x} & =f(x,u)+n \end{array}$$

(21) $$\begin{array}{cc} y & =Cx+m, \end{array}$$

where $f\left(\cdot \right):R^{16}\times R^{13}\mapsto R^{16}$ is a nonsmooth, nonlinear vector field and $C\in R^{2\times 16}$ is the output measurement matrix. Also considered are system noise $n\in R^{16}$ in Equation (20) and measurement noise $m\in R^{2}$ in Equation (21). The noise signals are assumed to be uncorrelated, mean free, white Gaussian processes with $E\left[nn^{T}\right]=Q_{n}$ and $E\left[mm^{T}\right]=R_{m}$. Figure 6 is a block diagram of the lower extremity dynamic model. Note that the input sEMG-signals to the diagram are the bandpass and linear envelope filtered signals that are generated from the raw sEMG measurements (the process of sEMG signal filtering is described in Section 3.2).

## 3. Methods and Material

This section presents the model-based filtering approach used to minimise the influence of modelling errors and measurement noise. In addition, the experimental setup and procedures used for the validation study are described. The filter of choice is cubature Kalman filter, since it increases numerical accuracy towards numerically problematic operations, such as the quadratic terms associated with the prediction of the covariance matrices [27].

### 3.1. Cubature Kalman Filter and Square-Cubature Kalman Filter

The *cubature Kalman filter* (CKF) was first presented in [27]. The CKF is different from, for example, the extended Kalman filter (EKF) or the unscented Kalman filter (UKF). A comparison between the CKF and UKF shows that the CKF is contained as a special case in the unscented transform [27]. However, in contrast to the UKF, the CKF does not use any negatively weighted sampling points and is, therefore, well suited for a numerically more stable implementation, the so-called square-root cubature Kalman filter (SCKF). The CKF is based on the nonlinear state-space model represented by a discretised version of Equations (20) and (21)

(22) $$\begin{array}{cc} x_{k+1} & =f_{d}\left(x_{k},u_{k}\right)+n_{k}, \end{array}$$

(23) $$\begin{array}{cc} y_{k} & =h_{d}\left(x_{k}\right)+m_{k} \end{array}$$

and on Bayes’ equations. In Equations (22) and (23), *k* denotes sampling instances of discrete time, $x_{k}$ is the discrete state vector and $y_{k}$ is the discrete measurement vector. The associated noise covariances $Q_{k}$ and $R_{k}$ are the discretised versions of the noise covariances in Equations (20) and (21). $f_{d}\left(\cdot \right):R^{n}\times R^{m}\mapsto R^{n}$ and $h_{d}\left(\cdot \right):R^{n}\mapsto R^{p}$ are nonlinear nonsmooth vector fields. For the prediction step the predictive probability density results in

(24) $$\begin{array}{cc} p(x_{k+1}|D_{k}) & =\int_{R^{n}}p\left(x_{k+1},x_{k}|D_{k}\right)dx_{k}\\ & =\int_{R^{n}}p\left(x_{k}|D_{k}\right)p\left(x_{k+1}|x_{k},u_{k}\right)dx_{k}. \end{array}$$

where $D_{k}={\left\{u_{i},y_{i}\right\}}_{i=1}^{k}$ denotes the input measurement pairs up to time *k* and *p* is the probability density. The posterior density is given by

(25) $$\begin{array}{cc} p(x_{k+1}|D_{k+1}) & =p(x_{k+1}|D_{k},u_{k+1},y_{k+1})\\ & =\frac{1}{c_{k+1}}p\left(x_{k+1}|D_{k},u_{k+1}\right)p\left(y_{k+1}|x_{k+1},u_{k+1}\right)\\ & =\frac{1}{c_{k+1}}p\left(x_{k+1}|D_{k}\right)p\left(y_{k+1}|x_{k+1}\right), \end{array}$$

where $c_{k}$ denotes the normalising constant, given by

(26) $$c_{k+1}=\int_{R^{n}}p\left(x_{k+1}|D_{k}\right)p\left(y_{k+1}|x_{k+1}\right)dx_{k+1}.$$

The observation density $p(y_{k+1}|x_{k+1})$ is determined by Equation (23). It should be noted that the multi-dimensional integrals of Equations (24) and (26) are not always solvable in complete form. However, for processes with a Gaussian probability distribution, Bayes’ equations can be brought to a recursive form, where only the expected value and the covariance are to be determined. The solution of the multidimensional integral is reduced to the form of *nonlinear function · Gaussian distribution*. For the expected value and the covariances, the following equations are obtained

(27) $$\begin{array}{cc} {\hat{x}}_{k+1|k} & =\int_{R^{n}}f_{d}\left(x_{k},u_{k}\right)N\left(x_{k};{\hat{x}}_{k|k},P_{k|k}\right)dx_{k}, \end{array}$$

(28) $$\begin{array}{cc} P_{k+1|k} & =\int_{R^{n}}f_{d}\left(x_{k},u_{k}\right)f_{d}^{T}\left(x_{k},u_{k}\right)N\left(x_{k};{\hat{x}}_{k|k},P_{k|k}\right)dx_{k}-{\hat{x}}_{k+1|k}{\hat{x}}_{k+1|k}^{T}+Q_{k}, \end{array}$$

(29) $$\begin{array}{cc} {\hat{y}}_{k+1|k} & =\int_{R^{n}}h_{d}\left(x_{k}\right)N\left(x_{k+1};{\hat{x}}_{k+1|k},P_{k+1|k}\right)dx_{k+1}, \end{array}$$

(30) $$\begin{array}{cc} P_{yy,k+1|k} & =\int_{R^{n}}h_{d}\left(x_{k+1}\right)h_{d}^{T}\left(x_{k+1}\right)N\left(x_{k+1};{\hat{x}}_{k+1|k},P_{k+1|k}\right)dx_{k+1}-{\hat{y}}_{k+1|k}{\hat{y}}_{k+1|k}^{T}+R_{k}, \end{array}$$

(31) $$\begin{array}{cc} P_{xy,k+1|k} & =\int_{R^{n}}x_{k+1}h_{d}^{T}\left(x_{k+1}\right)N\left(x_{k+1};{\hat{x}}_{k+1|k},P_{k+1|k}\right)dx_{k+1}-{\hat{x}}_{k+1|k}{\hat{y}}_{k+1|k}^{T}, \end{array}$$

where $N(x;\hat{x},P)$ denotes a Gaussian distribution with mean value $\hat{x}$ and variance P. The idea of the CKF is to approximate the integrals given in Equations (27)–(31) for the nonlinear vector fields with sets of weighted points. For these points $\xi_{i}$ and the weightings $\omega_{i}$, we ask for an arbitrary function $g\left(\cdot \right):R^{n}\mapsto R^{n}$ and an argument $x\in R^{n}$

(32) $$\int_{R^{n}}g\left(x\right)N\left(x;0,I\right)\approx \sum_{i=1}^{l}\omega_{i}g\left(\xi_{i}\right),$$

where I is the unity matrix of dimension *n*. In the CKF, the points are determined with a third-degree spherical-radial cubature rule. Then the points are given as

(33) $$\begin{array}{cc} \xi_{i} & =\sqrt{\frac{l}{2}}{\left[1\right]}_{i}, \end{array}$$

(34) $$\begin{array}{cc} \omega_{i} & =\frac{1}{l},\;i=1,2,\ldots l=2n, \end{array}$$

where ${\left[1\right]}_{i}$ denotes the *i*th point that is obtained by permutation and sign change of the components of the n-dimensional generator ${\left(1,0,\cdots ,0\right)}^{T}$ for the set of points

(35) $$\left[1\right]=\left\{\left[\begin{array}{c} 1\\ 0\\ \vdots \\ 0 \end{array}\right],\left[\begin{array}{c} 0\\ 1\\ \vdots \\ 0 \end{array}\right],\cdots \left[\begin{array}{c} 0\\ 0\\ \vdots \\ 1 \end{array}\right],\left[\begin{array}{c} -1\\ 0\\ \vdots \\ 0 \end{array}\right],\left[\begin{array}{c} 0\\ -1\\ \vdots \\ 0 \end{array}\right],\cdots \left[\begin{array}{c} 0\\ 0\\ \vdots \\ -1 \end{array}\right]\right\}.$$

The integrals given in Equations (27)–(31) can be solved with the Equations (32)–(34) for Gaussian distributions. The resulting CKF algorithm is according to the UKF algorithm [52], with the exception of the CKF cubature sampling points $X_{i}$ which are chosen in a different way as

(36) $$\begin{array}{cc} X_{i} & =\left\{\begin{array}{cc} 0 & \mathrm{for}\;i=0\\ \sqrt{P_{x}}\xi_{i}+\hat{x} & \mathrm{for}\;i=1,\ldots ,2n \end{array}\right. \end{array}$$

(37) Wi(m)=Wi(c)=0Pxξi+x^fori=012nfori=1,…,2n.

Comparison of the sigma points of the UKF and the cubature points of the CKF shows that the CKF is a special case of the UKF [27]. However, in contrast to the unscented transformation, the CKF gives a mathematical justification for an accurate sampling of the weighted Gaussian integrals. To avoid numerical difficulties associated with limited numerical precision, the CKF is formulated in its SCKF version that avoids computation of the squareroot of the covariance matrices (positive definiteness of covariance matrices is ensured). In the following, for convenience, the SCKF is presented in a compact form.

The SCKF is initialised with $S_{0|0}$ that denotes the root factor of the error covariance $P_{k|k}=S_{k|k}S_{k|k}^{T}$. In the filter prediction phase, the cubature points are determined and propagated through the state equation and the predicted state is estimated

(38) $$\begin{array}{cc} X_{i,k|k} & =S_{k|k}\xi_{i}+{\hat{x}}_{k|k},\;i=1,\ldots ,2n, \end{array}$$

(39) $$\begin{array}{cc} X_{i,k+1|k}^{*} & =f_{d}\left(X_{i,k|k},u_{k}\right),\;i=1,\ldots ,2n, \end{array}$$

(40) $$\begin{array}{cc} {\hat{x}}_{k+1|k} & =\frac{1}{2n}\sum_{i=1}^{2n}X_{i,k+1|k}^{*}. \end{array}$$

The prediction of the error covariance and accordingly its root factor $S_{k+1|k}$ with $P_{k+1|k}=S_{k+1|k}S_{k+1|k}^{T}$ follows a triangularisation(S=tria(A) denotes the triangularisation of the matrix A, with e.g., a QR-decomposition)

(41) $$S_{k+1|k}=\mathrm{tria}\left(\left[X_{k+1|k}^{*}\;S_{Q,k}\right]\right),$$

with $Q_{k}=S_{Q,k}S_{Q,k}^{T}$ and moreover

(42) $$X_{k+1|k}^{*}=\frac{1}{\sqrt{2n}}\left[X_{1,k+1|k}^{*}-{\hat{x}}_{k+1|k}\;X_{2,k+1|k}^{*}-{\hat{x}}_{k+1|k}\;\cdots \;X_{2n,k+1|k}^{*}-{\hat{x}}_{k+1|k}\right].$$

The first steps of the filter correction phase can be formulated analogous to the filter prediction phase

(43) $$\begin{array}{cc} X_{i,k+1|k} & =S_{k+1|k}\xi_{i}+{\hat{x}}_{k+1|k},\;i=1,\ldots ,2n, \end{array}$$

(44) $$\begin{array}{cc} Y_{i,k+1|k} & =h_{d}\left(X_{i,k+1|k}\right),\;i=1,\ldots ,2n, \end{array}$$

(45) $$\begin{array}{cc} {\hat{y}}_{k+1|k} & =\frac{1}{2n}\sum_{i=1}^{2n}Y_{i,k+1|k}, \end{array}$$

(46) $$\begin{array}{cc} S_{yy,k+1|k} & =\mathrm{tria}(\left[Y_{k+1|k}\;S_{R,k}\right]) \end{array}$$

with $R_{k}=S_{R,k}S_{R,k}^{T}$ and

(47) $$Y_{k+1|k}=\frac{1}{\sqrt{2n}}\left[Y_{1,k+1|k}-{\hat{y}}_{k+1|k}\;Y_{2,k+1|k}-{\hat{y}}_{k+1|k}\;\cdots \;Y_{2n,k+1|k}-{\hat{y}}_{k+1|k}\right]\;.$$

The cross-covariance is estimated explicitly (not as a root factor)

(48) $$P_{xy,k+1|k}=X_{k+1|k}Y_{k+1|k}^{T},$$

where

(49) $$X_{k+1|k}=\frac{1}{\sqrt{2n}}\left[X_{1,k+1|k}-{\hat{x}}_{k+1|k}\;X_{2,k+1|k}-{\hat{x}}_{k+1|k}\;\cdots \;X_{2n,k+1|k}-{\hat{x}}_{k+1|k}\right].$$

The Kalman gain matrix and the innovation are calculated as (The operator/denotes right-hand side matrix divisions. Thus, B/A is short for the solution of xA=B with a least squares approach).

(50) $$\begin{array}{cc} K_{k+1} & =\left(P_{xy,k+1|k}/S_{yy,k+1|k}^{T}\right)/S_{yy,k+1|k}, \end{array}$$

(51) $$\begin{array}{cc} {\tilde{y}}_{k+1} & =y_{k+1}-{\hat{y}}_{k+1|k}, \end{array}$$

where finally state and error covariance root factor update is given by

(52) $$\begin{array}{cc} {\hat{x}}_{k+1|k+1} & ={\hat{x}}_{k+1|k}+K_{k+1}{\tilde{y}}_{k+1}, \end{array}$$

(53) $$\begin{array}{cc} S_{k+1|k+1} & =\mathrm{tria}(\left[X_{k+1|k}-K_{k+1}Y_{k+1|k}\;K_{k+1}S_{R,k}\right]). \end{array}$$

### 3.2. Experimental Setup

**Figure 7:**
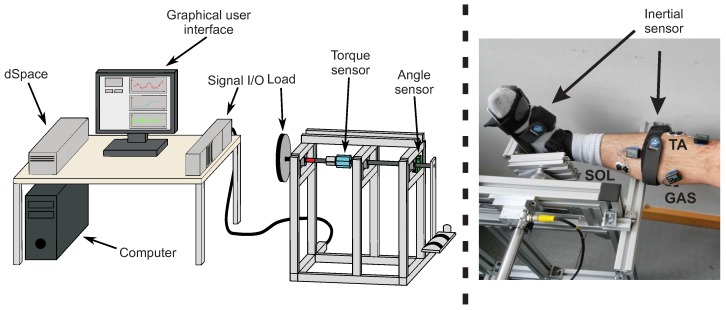
Overview of the test-bench used to validate ankle joint stiffness estimator, with torque sensor (blue), angle sensor (green) and optional integration of an actuator (red) (**left**). The photograph (**right**) shows a a test person with body-worn sensors.

The experimental setup consists of body-worn sensor systems and a custom built test-bench. Two synchronised sensor systems were used that were attached to the body of a test person. A surface electromyography (EMG) measurement was conducted with the MyoMuscle system (Noraxon Inc., Scottsdale, AZ, USA) following the SENIAM (Surface ElectroMyoGraphy for the Non-Invasive Assessment of Muscles) guidelines to place the electrodes [53]. Segmental orientation was measured with nine-degrees-of-freedom (9DOF) magnetic-angular-rate-gravity (MARG) sensors of the MyoMotion system (Noraxon Inc., Scottsdale, AZ, USA). Both systems consist of wireless sensor nodes attached to the test person, that allow the user to move freely during stiffness determination tests. The sensor systems allow for synchronised recordings with a standard computer (MyoResearch software, Noraxon Inc., Scottsdale, AZ, USA). sEMG signals are sampled at a sampling frequency of 1500 Hz (16 bit analog-digital conversion) and low-pass filtered (corner frequency 500 Hz), as well as high-pass filtered (corner frequency 10 Hz). The segmental orientation angles with respect to an inertially fixed reference system are provided with a sampling frequency of 100 Hz. The experimental setup is shown in Figure 7 and consists of a personal computer with data acquisition and real-time operating system (DS1104, dSpace GmbH, Paderborn, Germany) and test-bench integrated sensors. These sensors are mounted to the rotational axis of the test-bench and are used to determine the torque (DR-2477, Lorenz Messtechnik GmbH, Alfdorf, Germany) and the rotational angle (HDmag MHAD 50, Baumer Group, Fraunfeld, Switzerland). For the determination of a joint torque, a known axial reference inertia is employed in combination with a nonlinear dynamic model. The parameters of this nonlinear dynamic model were determined in an experimental identification procedure, described in [54]. Thus, by subtracting all of the test-bench torques from the torque sensor signal, the human joint torque that acts externally on the test-bench can be extracted and used as a reference for the filter validation. Finally, the neuromusculoskeletal model and the SCKF were implemented in Matlab (The MathWorks, Natick, MA, USA).

## 4. Simulation and Experimental Results

The model-based stiffness estimator was validated in simulations and in an experiment including five test persons. However, for the in silico tests the model was parameterised with values from literature [15,43,44,55]. Further parameters were taken from [28]. A table with model parameters and sources is given in the Appendix D, Table A3 and Table A4.

### 4.1. Simulation Study

**Figure 8:**
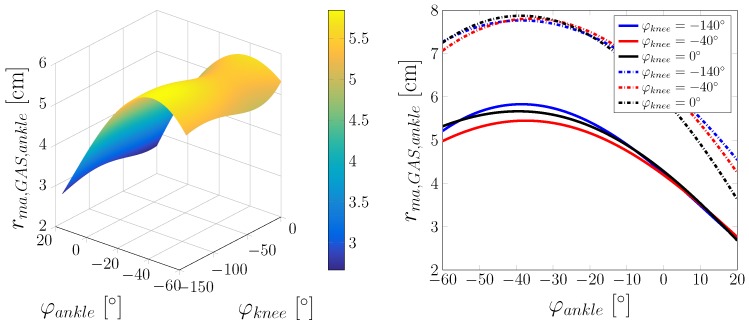
Lever arm of the GAS at the ankle joint. **Left**: Lever arm dependent on $\varphi_{ankle}$ and $\varphi_{knee}$. **Right**: Lever arm depending on $\varphi_{ankle}$ at different values of $\varphi_{knee}$ (solid lines: lever arms determined via the distance to the joint centre; dashed lines: lever arms calculated with the principle of virtual displacements).

Simulation tests were conducted in static and dynamic situations. In the static case, the focus of investigation lies in the varying length and lever arms of single muscles that are investigated depending on the kinematics of the lower extremity model. Of interest are the biarticulate muscles, also with regard to changes in the neighbouring joints. In case of the ankle joint, this is the GAS. Figure 8 shows the lever arm of the GAS with respect to the ankle joint.

In addition to the lever arms that were calculated via the distance to the joint centre, lever arms that were calculated with the principle of virtual displacements are shown, where for the torque

(54) $$F_{i}^{T}\partial l_{MT_{i}}=\tau_{i,j}^{T}\partial \varphi_{j},$$

the lever arm of the muscle *i* at the joint *j* can be stated as

(55) $$r_{ma_{i,j}}=\frac{\partial l_{MT_{i}}}{\partial \varphi_{j}}.$$

**Figure 9:**
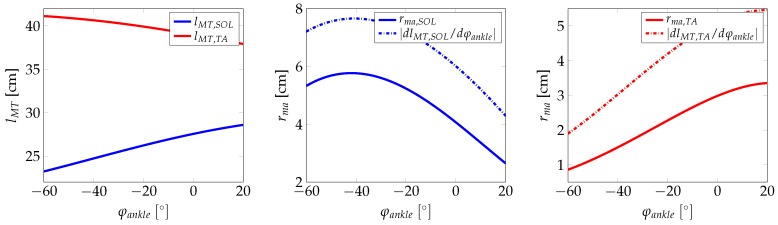
MTC of the TA and the SOL (**left**), lever arm of the SOL (**middle**) and lever arm of the TA (**right**).

The lever arm calculated from the principle of virtual displacements shows an offset when compared to the lever arms that were calculated from the model. On the one hand, these differences might be due to anatomical limitations in the attachment of the muscles and/or the reduction to the two-dimensional case. On the other hand, the fact that the lever arm is calculated via a rotation with a constant generalised coordinate axis might be the reason for the differences in the observed lever arm. However, the main shape of the two lever arms curves are similar. Other than the GAS, the ankle joint contains the uniarticular muscles SOL and TA for which the muscle and the lever arms are shown in Figure 9. As before, the model-calculated lever arms are compared to lever arms computed with the principle of virtual displacement.

**Figure A1:**
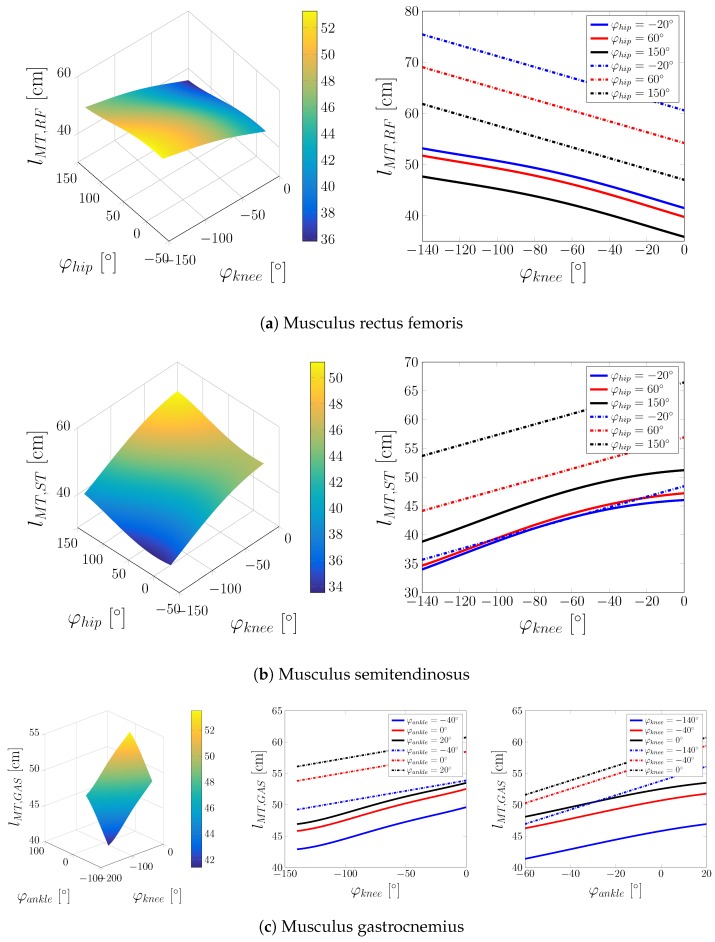
Muscle-tendon length *l_MT_* of the considered biarticulate muscles. **Left**: Length in dependence of the angle of the two joints, which the muscle acts on. **Middle/right**: Length depending on the angle of the neighbouring joint at different fixed values for the neighbouring joint angles (solid lines: length from the proposed model, dashed lines: length after [61]).

For the other muscles acting on the knee, analogous results are obtained. Moreover, the muscle length and lever arm values are in accordance with published values [56,57,58]. In the Appendix A, Figure A1 compares the muscle length to data from the literature. We conclude that a hinge model approach is sufficient for ankle joint modelling. After static simulation tests, the SCKF was verified in dynamic in silico tests. For that, the model of Equations (20) and (21) was discretised at a sampling time of $T_{s}$ with the resulting discrete state-space equations

(56) $$\begin{array}{cc} x_{k+1} & =x_{k}+T_{s}f\left(x_{k},u_{k}\right)+n_{k} \end{array}$$

(57) $$\begin{array}{cc} y_{k} & =Cx_{k}+m_{k}, \end{array}$$

where $n_{k}$ and $m_{k}$ are the corresponding discretised versions of the system and measurement noise processes. In the simulation model, in comparison to the filter model, additive white Gaussian noise is added to the states and to the measurements. Moreover, the external hip velocity and acceleration are obtained from the hip position by means of differentiation (a low pass with relatively high crossover frequency is used to limit to the influence of high frequency noise). This filter is thereby implemented by using the following transfer function

(58) $$G\left(s\right)=\frac{s}{\left(\frac{s}{30}+1\right){\left(\frac{s}{300}+1\right)}^{2}}.$$

**Figure 10:**
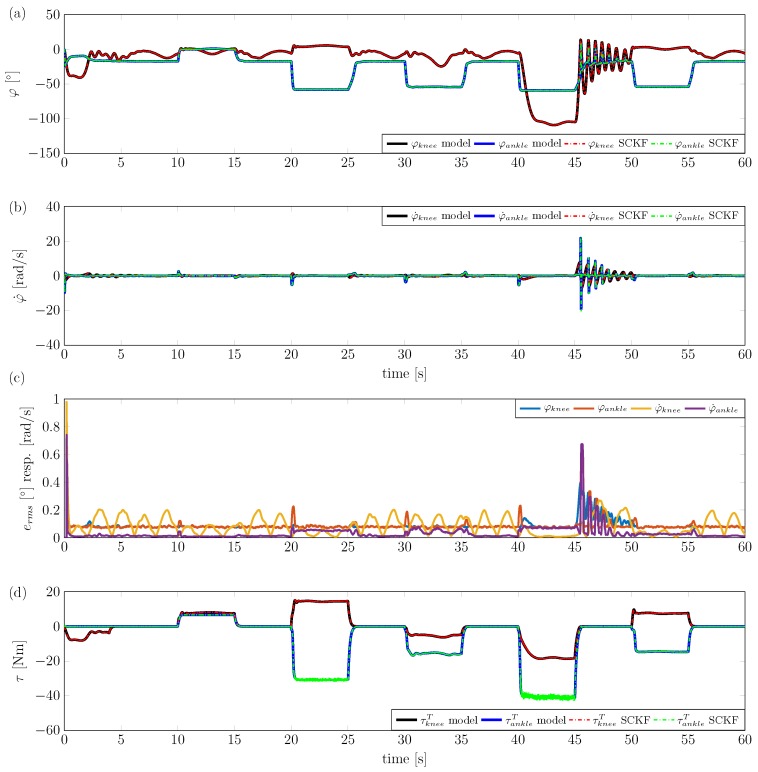
Application of the square-root cubature Kalman filter (SCKF) to the sagittal plane lower extremity kinematic model. $\varphi_{hip}=20^{\circ }\cdot \sin\left(\frac{\pi }{2}\;\mathrm{rad}/s\;\cdot t\right)$. (**a**) Estimation of knee and ankle angle. (**b**) Estimation of knee and ankle angular velocity. (**c**) RMS errors of angle and angular velocity estimations. (**d**) Estimation of knee and ankle torques.

To test the stiffness estimation in an appropriate dynamic situation, all three angles (hip, knee and ankle) are initialised with an initial state of 0. Note that the hip flexion-extension angle is important for the full-order model for knee and ankle. The muscles that are considered in the lower extremity model are then activated in different patterns over a time window of 5 s, followed by a resting phase of 5 s. Figure 10 presents an example of the SCKF for the full-order nonlinear model.

During the experiment, the hip angle is changed in a sinusoidal manner with an amplitude of $20^{\circ }$ and a frequency of $\frac{\pi }{2}\;\mathrm{rad}/s$. Filter internal states are used to calculate an overall ankle and knee torque, which is compared to the torque of the lower extremity model. As can be seen in Figure 10, the filter shows good state estimation performance, with slightly larger errors in the angular rates compared to the angles. Angular errors (RMS) remain below $0.4^{\circ }$ and angular rate errors (RMS) remain below 0.7rad/s. Ankle and knee torques are estimated quite accurately, also during dynamic situations (e.g., during muscle activation).

### 4.2. Experimental Study

The substantial extension of the lower extremity dynamic model is the ankle model. To validate the ankle joint stiffness estimator under real-world experimental conditions, the test-bench described in Section 3.2 was used to provide reference measurements for ankle joint stiffness and angle. Before the measurement, Ag/AgCl surface electrodes were placed on the corresponding muscles and body-worn sensors of the MyoMuscle system were attached to a test person. In addition, the orientation of foot, shank and thigh was determined by applying 9DOF MARG sensor nodes of the MyoMotion system with straps to the respective segments. The lower extremity SCKF filter model had to be adapted to the experimental setup. Due to a connection of the ankle joint rotational axis with the rotational axis of the test-bench, there are no ground reaction forces. Furthermore, the torque sensor, used in the test-bench, measures additional external torques from the reference mass and viscous friction. These external torques were determined by employing a nonlinear dynamic model of the test-bench and subtracted from the sensor signal [28,54]. Finally, the dynamic model of the SCKF was reduced by assuming constant knee and hip angle values $q_{2}=\varphi_{knee}$ and $q_{1}=\varphi_{hip}$, respectively. This simplification is assumed to be valid, since the test persons were instructed to sit upright and not to move their knee or their hip. Thus, in the model ${\dot{q}}_{1}={\ddot{q}}_{1}={\dot{q}}_{2}={\ddot{q}}_{2}=0$ is assumed with subsequent simplifications in the model dynamics.

**Figure 11:**
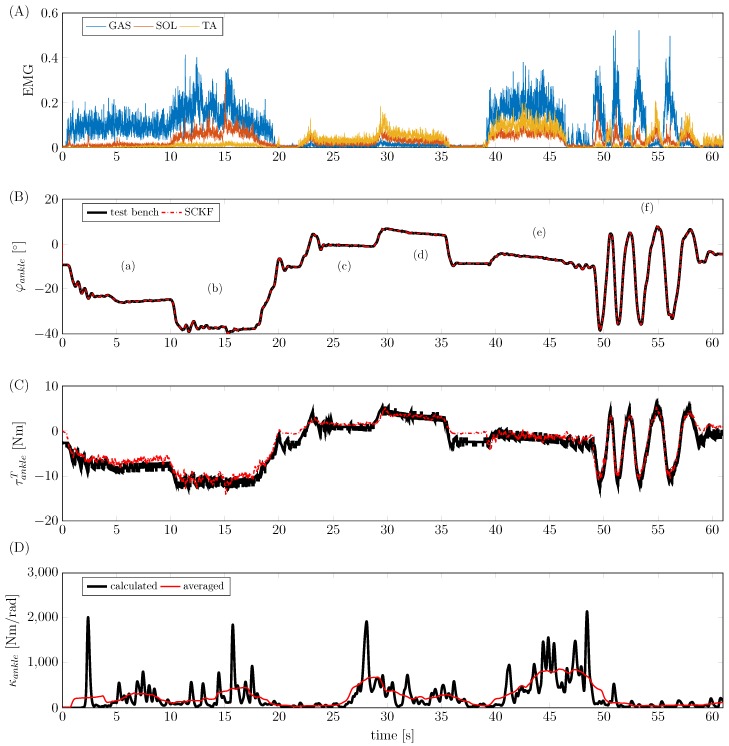
Exemplary results of the experimental validation of test person ID004. (**A**) Normalized, rectified and averaged EMG input signals. (**B**) Estimation of ankle angle with (**a**) plantar flexion; (**b**) maximum plantar flexion; (**c**) dorsiflexion; (**d**) maximum dorsiflexion; (**e**) coactivation of flexors and extensors; and (**f**) dynamic plantar flexion/dorsiflexion. (**C**) Estimation of ankle torque. (**D**) Estimation of ankle stiffness.

**Figure 12:**
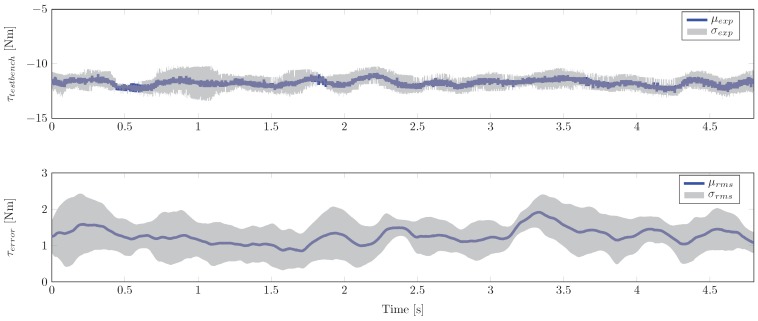
Statistical results for the test persons at maximum plantar flexion. **Upper figure**: Average mean and standard deviation of test-bench torque measurements. **Lower figure**: Average mean and standard deviation of RMS errors between estimation test-bench reference.

After model parameterisation and preparation of the experimental setup, a study with five male test persons was conducted (age [years]: 29±5.7, height [cm]: 181.6±1.82, weight [kg]: 79.2±8.58). During the experiments, the test persons were instructed to conduct a number of different extensions/flexions after an initial maximum voluntary contraction test of muscles with relevance to the ankle joint. sEMG signals were then normalised to maximum voluntary contraction before they were applied to the filter. Characteristic experimental movements included plantar flexion and dorsiflexion, as well as coactivation of flexors and extensors. An overview of the experimental test protocol is given in the Appendix B (Table A1). An example of such an experiment with test person ID004 is shown in Figure 11; as can be seen, the SCKF is able to reconstruct the ankle torque based on surface EMG and segmental orientation measurements. Ankle joint torques of up to 12 Nm were generated during the measurements. Moreover, the joint torque estimation is accurate for muscle coactivation (phase (e) in Figure 11) as well as a dynamic cyclic movement (phase (f) in Figure 11). In addition to the model-based estimation of the ankle joint torque, the quasi-stiffness $\kappa_{ankle}$ was computed from the model states

(59) $$\begin{array}{cc} \kappa_{ankle} & =\frac{\partial \tau_{ankle}^{T}}{\partial \varphi_{ankle}}=\frac{\partial }{\partial \varphi_{ankle}}\left(\sum_{i=1}^{N}r_{ma_{i,ankle}}F_{i}^{T}\right)\\ & =\frac{\partial r_{ma_{ankle}}}{\partial \varphi_{ankle}}F_{ankle}^{T}+r_{ma_{ankle}}\frac{\partial F_{ankle}^{T}}{\partial \varphi_{ankle}}, \end{array}$$

where $r_{ma_{ankle}}={\left[r_{ma_{1,ankle}},r_{ma_{2,ankle}},\ldots ,r_{ma_{N,ankle}}\right]}^{T}$ and $F_{ankle}^{T}={\left[F_{1}^{T},F_{2}^{T},\ldots ,F_{N}^{T}\right]}^{T}$ denote the lever arms and the forces of the muscles that were considered in the model, respectively. In the resulting stiffness in Figure 11, peaks on the ankle joint stiffness $\kappa_{ankle}$ can be observed that are of unphysiological value and dynamics. We assume that the noisy sEMG signals which are given to the contraction dynamics are responsible for the high dynamics in the observed stiffness values. If an average mean stiffness value is determined, stiffness values show good agreement with published values [59,60]. For a quantitative investigation of the estimation quality, a statistical analysis was conducted. The results (Figure 12) show the deviation of the estimated filter torque from the reference torque of the test-bench at maximum plantar flexion. The average RMS error is 1.27 Nm. In addition to these results, in the Appendix C, Table A2 lists the estimation errors obtained for the different test persons.

## 5. Discussion and Conclusions

The goal of this study was to develop a novel model-based estimator for the determination of ankle joint stiffness, employing body-worn sensors only. For this, an existing sagittal plane dynamic model of the human knee was extended to describe a full sagittal plane model of the lower extremity. The dynamic model is thus driven by a measured hip angle (and corresponding time derivatives), where ankle and knee torques are estimated based on sEMG and segmental orientation data. To minimise the influence of measurement noise and model errors that would occur in a forward simulation, a nonlinear state-observer was developed. The filter of choice was the square-root version of the CKF because of its ability to handle nonsmooth, highly nonlinear dynamic systems, and no necessity to process Cholesky decompositions of the covariance matrix. The SCKF was able to reconstruct knee and ankle joint torque/quasi-stiffness in simulations, as well as ankle joint torque/quasi-stiffness in an experimental series with five test persons. However, in different ankle joint dynamic experiments a decreasing estimation performance was observed. Since this decrease in the performance was non-consistent with multiple measurements within the group of test persons, a general observer problem for measurements with higher dynamics can be excluded. An improved test bench is required to further investigate observed effects. In the current setup, the rotation axis of the test-bench is fixed, whereas the axis of the TC is moving. This might lead to involuntary inversion/eversion movements that could be responsible for the individually observed errors. Moreover, the estimation of the ankle joint stiffness shows unphysiologically high peaks, whereas the average mean of the estimated ankle joint stiffness lies within the physiological range. An obvious explanation for this effect is a noisy sEMG measurement, including movement artifacts. An additional explanation might the uncertainty in the muscle model time constants that lead to activation mismatches. To improve the performance of the model one option would be segmental parameterisation of a certain subset of model parameters. A combination of segmental parameterisation and calibration procedures, as described in [14,20], might improve the quality of our model. Moreover, a comparison of an extended model with elastic tendons to our model (concentrated visco-elastic elements) should be investigated. We are currently investigating these items, as well as application of the full-order observer for combined knee/ankle stiffness estimation.

## Appendix B. Experimental Protocol

**Table A1:** Overview of the experimental protocol.

	Movement	Description	Approximate Duration (s)
0	Maximum voluntary contraction	Maximum voluntary contraction of involved muscles	5
1	Plantar flexion	Loose stretching of the foot	5
2	Maximal plantar flexion	Stretching the foot as far as possible	5
3	Dorsiflexion	Loose upward flexion of the foot	5
4	Maximal dorsiflexion	Flexing the foot upward as far as possible	5
5	Coactivation	Tensing of all muscles around the ankle, thereby locking foot in a fixed position	10
6	Dynamic plantar/dorsiflexion	Dynamic switching between stretching and upward flexion	10

## Appendix C. Experimental Performance

**Table A2:** Overview of the estimation errors obtained during the experiments.

ID	Peak Error (Nm)	Test-Bench Torque at Peak (Nm)	Peak Error Excluding Dynamic Movement (Nm)	Test-Bench Torque at Peak Excluding Dynamic Movement (Nm)
001	6.7238	−11.0530	3.6368	10.5838
002	6.4308	12.3142	4.6228	21.2717
003	5.6401	12.4584	5.6401	12.4584
004	4.0512	−4.6143	4.0512	−4.6143
006	8.1544	22.45	8.1544	22.45

## Appendix D. Model Parameters

**Table A3:** Model parameters I.

Parameter	Descriptionri	Value	Source
$\alpha_{RF}$	Pennation angle of RF	13.9 ${}^{\circ }$	[55]
$\alpha_{HAMS}$	Pennation angle of HAMS, represented here by the semimembranosus	15.1 ${}^{\circ }$	[55]
$\alpha_{GAS}$	Pennation angle of GAS, represented here by the medial head of GAS	9.9 ${}^{\circ }$	[55]
$\alpha_{VM}$	Pennation angle of VM	29.6 ${}^{\circ }$	[55]
$\alpha_{SOL}$	Pennation angle of SOL	28.3 ${}^{\circ }$	[55]
$\alpha_{TA}$	Pennation angle of TA	9.6 ${}^{\circ }$	[55]
$v_{M,max_{RF}}$	Maximum contraction speed of RF	0.76 m/s	[55]
$v_{M,max_{HAMS}}$	Maximum contraction speed of HAMS, represented here by the semimembranosus	0.69 m/s	[55]
$v_{M,max_{GAS}}$	Maximum contraction speed of GAS, represented here by the medial head of GAS	0.51 m/s	[55]
$v_{M,max_{VM}}$	Maximum contraction speed of VM	0.97 m/s	[55]
$v_{M,max_{SOL}}$	Maximum contraction speed of SOL	0.44 m/s	[55]
$v_{M,max_{TA}}$	Maximum contraction speed of TA	0.68 m/s	[55]
$l_{thigh}$	Length of thigh	individual	Reg. Equations from [62]
$l_{shank}$	Length of shank	individual	Reg. Equations from [62]
$l_{foot}$	Length of foot	individual	Reg. Equations from [62]
$l_{thigh_{m}}$	Distance from distal end of thigh to its centre of mass	individual	Reg. Equations from [47]
$l_{shank_{m}}$	Distance from distal end of shank to its centre of mass	individual	Reg. Equations from [47]
$l_{dorsum}$	Length of dorsum	individual	Based on [46]
$l_{foot_{m,x}}$	x coordinate of centre of mass of foot with respect to the foot reference frame	individual	Based on [46]
$l_{foot_{m,y}}$	y coordinate of centre of mass of foot with respect to the foot reference frame	individual	Based on [46]
$m_{thigh}$	Mass of thigh	individual	Reg. Equations from [47]
$m_{shank}$	Mass of shank	individual	Reg. Equations from [47]
$m_{foot}$	Mass of foot	individual	Reg. Equations from [47]
$I_{thigh}$	Principal moments of inertia of thigh	individual	Reg. Equations from [47]
$I_{shank}$	Principal moments of inertia of shank	individual	Reg. Equations from [47]
$I_{foot}$	Principal moments of inertia of foot	individual	Reg. Equations from [47]

**Table A4:** Model parameters II.

Parameter	Descriptionri	Value	Source
$\alpha_{TC_{fml}}$	Angle between talocrural joint axis and midline of foot, projected onto the horizontal plane	84 ${}^{\circ }$	[34]
$\alpha_{TC_{tla}}$	Angle between talocrural joint axis and long axis of tibia	80 ${}^{\circ }$	[34]
*a*	Principle axis length of the femur condyle ellipse	3.31 cm	[63]
*b*	Semi-axis length of the femur condyle ellipse	2.71 cm	[63]
$\alpha_{fc}$	Angle between femur axis and femur condyle semi-axis	14 ${}^{\circ }$	[63]
$l_{tp}$	Tibia plateau length	5.3 cm	[63]
$\alpha_{tp}$	Angle between perpendicular with respect to tibia axis and tibia plateau	3 ${}^{\circ }$	[63]
$l_{tt}$	Distance between tibia plateau edge and tibial tuberosity	4.5 cm	[63]
$\alpha_{tt}$	Angle between tibia axis and tibial tuberosity appendage	15 ${}^{\circ }$	[63]
$l_{pl}$	Patella tendon length	6.5 cm	[63]
$l_{p}$	Patella length	3.94 cm	[64]
$t_{p}$	Patella width	1.63 cm	[64]
$p_{O,SOL}^{shank}$	Coordinates of origin of SOL in shank reference frame [cm]	[−2.92, 24.67, 0.06]	[43]
$p_{I,SOL}^{foot}$	Coordinates of insertion of SOL in foot reference frame [cm]	[−3.65, −2.88, 0.56]	[43]
$p_{I,GAS}^{foot}$	Coordinates of insertion of GAS in foot reference frame [cm]	[−3.68, −2.89, 0.28]	[43]
$p_{O,TA}^{shank}$	Coordinates of origin of TA in shank reference frame [cm]	[−1.55, 21.75, 1.34]	[43]
$p_{O_{eff},TA}^{shank}$	Coordinates of effective origin of TA in shank reference frame [cm]	[2.56, 2.57, −0.93]	[43]
$p_{I,TA}^{foot}$	Coordinates of insertion of TA in foot reference frame [cm]	[18.50, −5.10, −3.30]	[43]
$p_{I_{eff},TA}^{foot}$	Coordinates of effective insertion of TA in foot reference frame [cm]	[7.57, −4.20, −1.96]	[44]
$F_{opt,i}^{M}$	Maximum isometric force of muscle *i*	individual	Exp. det.
	Parameters of passively generated viscoelastic torques	individual	Exp. det.
